# Pure Instinct

**Published:** 1889-08

**Authors:** 


### PURE INSTINCT.

During the hot summer months, the "mud wasp" builds mud cells in clusters of from two to four for its offspring, each cell being precisely the size of the full grown chrysalis, from which the young emerges in due course of development in the early summer ; and yet the egg from which the young is produced is hardly larger than the point of a pin. When the cell is about completed, the egg is laid at its further extremity, and the cavity usually filled with spiders, then it is finally closed for the winter ; the parent wasps dying off, and never living to see their young emerge. The mud cells are always placed where it is dry, usually under a roof, secure from rain and snow, as absolute dryness is essential to the development of the young. The spiders are placed in the cavity as food for the larva during its growth. This process has been going on for centuries, and still continues, without the mud wasp ever seeing its offspring. Is this a case where instinct is superior to reason, or is it an evidence of the working of a higher power than either reason or instinct ? Would reason continue to operate for thousands of years, without seeing the slightest result ? It cannot be supposed that the mud wasp knows anything about its offspring, or that it will ever have any.—"GREELEY."

We have often observed the systematic labors of the mud wasp, in building its cells, and catching and entombing therein, numbers of live spiders, to serve as food for its young. The spider was always of the same genus as if in becoming food for the young wasp it subserved a natural destiny, having in it a smack of retributive justice.

But after all is not this the way of life in its entirety ? The weak are the victims of the strong, the world over, nor is man any exception to the rule.—ED.

